# Accuracy of VO_2_ max Estimates From Apple Watch Series 10

**DOI:** 10.1016/j.mcpdig.2026.100357

**Published:** 2026-04-07

**Authors:** Rory Lambe, Moritz Schumann, Lauren Donnelly, Sherene Hamilton, Saoirse Lally, Eve Rafter, Seán O’Reilly, Thomas White, Cailbhe Doherty

**Affiliations:** a School of Public Health, Physiotherapy and Sports Science, University College Dublin, Dublin, Ireland; b Insight Research Ireland Centre for Data Analytics (R.L., C.D.), University College Dublin, Dublin, Ireland; c Department of Health and Sport Sciences, Technical University of Munich, Munich, Germany

## Abstract

**Objective:**

To assess the validity of maximal oxygen uptake (VO_2_ max) estimates from Apple Watch Series 10 through comparison with indirect calorimetry, the criterion method.

**Participants and Methods:**

Participants completed a maximal exercise treadmill test using indirect calorimetry in accordance with the modified Åstrand protocol. They subsequently generated a VO_2_ max estimate with Apple Watch Series 10. The agreement between Apple Watch and indirect calorimetry was assessed using Bland-Altman analysis, mean absolute error, and mean absolute percentage error. Data were collected between October 2024 and January 2025.

**Results:**

Forty healthy adults (20 [50%] women) completed exercise testing. Apple Watch underestimated VO_2_ max overall, with wide limits of agreement indicating measurement variability (mean difference, –6.25 mL/kg per minute [95% CI, –8.28 to –4.23]; limits of agreement, –18.23 to 5.73). The mean absolute error was 6.79 mL/kg per minute (95% CI, 4.91-8.67), and the mean absolute percentage error was 13.2% (95% CI, 9.6-16.8). Based on reference standards from the Fitness Registry and the Importance of Exercise National Database, Apple Watch correctly classified the cardiorespiratory fitness percentile of 5 (14%) participants and classified a further 12 (34%) within 1 adjacent percentile band of their actual band.

**Conclusion:**

Apple Watch VO_2_ max estimates showed poor individual-level agreement with criterion measures, although our findings suggested utility for population-level assessment in research trials. Longitudinal validation of measurement reliability is needed to evaluate use as an alternative to conventional submaximal prediction methods.

## Graphical abstract

Graphical abstract providing a study overview.

**Figure:**
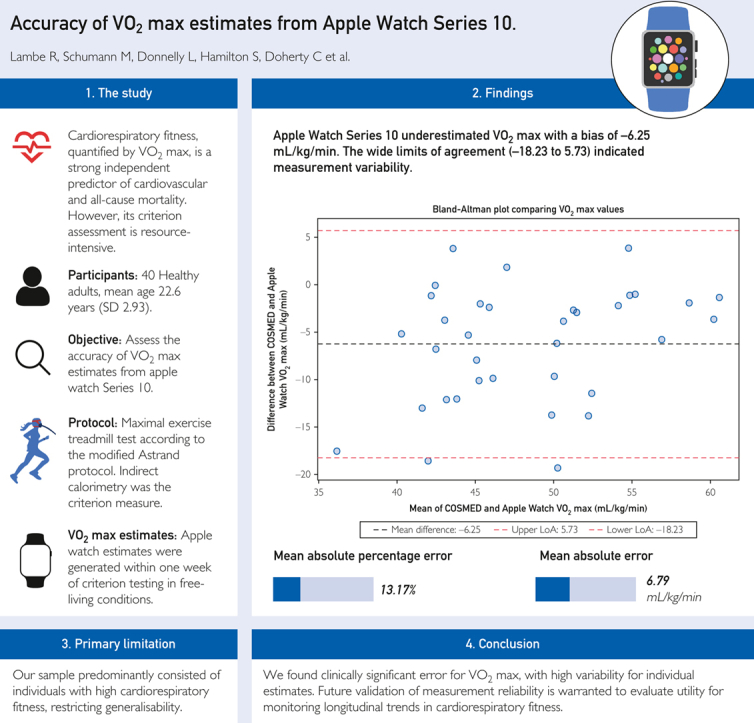

Cardiorespiratory fitness, quantified by maximum oxygen uptake (VO_2_ max), is a strong independent predictor of all-cause mortality, with a higher prognostic value for cardiovascular events than conventional risk factors, including hypertension and hypercholesterolemia.^1^, ^2^, ^3^ It has been associated with cancer and type 2 diabetes risk,^2^^,^^4^ and in recognition of its significance, the American Heart Association has advocated for its adoption as a clinical vital sign.^5^

Despite this, direct assessment of VO_2_ max remains infrequent in routine care, as indirect calorimetry is time-consuming, costly, and unsuitable for patients who cannot exercise at maximal intensity.^6^ As an alternative, VO_2_ max is often estimated from submaximal exercise using prediction equations, particularly among patients with cardiovascular disease. Although pragmatic, submaximal predictions are prone to clinically significant error,^7^, ^8^, ^9^ and assessments can be infrequent due to the resource constraints of health care professionals.

The ubiquity of consumer wearables, such as Apple Watch, presents an opportunity for longitudinal, self-directed monitoring of cardiorespiratory fitness. Apple Watch estimates VO_2_ max from heart rate response to submaximal exercise, much like traditional submaximal prediction methods.^10^ Its machine learning algorithm also combines age, sex, height, weight, and Global Positioning System (GPS) data to generate a prediction.^10^ This scalable method could enable chronic disease monitoring beyond clinical environments and provide digital end points in large-scale research trials.^11^^,^^12^ Conversely, inaccurate data may misrepresent physiological status, compromising research and clinical decision-making.

The myriad factors that affect measurements render accurate estimation challenging. These include physiological factors such as individual variation in heart rate response to exercise, environmental factors such as skin contact and motion,^13^ and external influences such as caffeine intake and carrying additional weight—in the form of bags, for instance.^10^ As Apple Watch estimates are derived from unstructured exercise, it is difficult to control for these factors. In addition, iterative software and hardware updates necessitate recurrent validation across device generations.^14^, ^15^, ^16^

Validation of Apple Watch VO_2_ max estimates remains sparse. Both studies published to date found that Apple Watch underestimated VO_2_ max.^17^^,^^18^ However, the existing literature is limited by small sample sizes, inconsistent adherence to best practice validation guidelines and assessment of Apple Watch models that have now been discontinued. Robust validation of contemporary devices is needed.

Here, we evaluated VO_2_ max estimates from Apple Watch Series 10 against indirect calorimetry to inform their use in clinical, research, and personal health contexts. This study aimed to quantify the level of agreement and the margin of error between the two methods (see Graphical Abstract).

## Participants and Methods

### Study Design and Oversight

This cross-sectional validation study was conducted in Dublin, Ireland, between October 2024 and January 2025. We recruited a purposive convenience sample that was sex-balanced. Healthy adults, aged 20 years or older, were recruited from the Dublin area via posters, email, and word of mouth. This included university staff and students, recreational athletes, and individuals who were physically inactive. Exclusion criteria included cardiovascular disease diagnosis or use of medication affecting cardiovascular function. All participants completed the Physical Activity Readiness Questionnaire Plus and provided written informed consent. Ethical approval was granted by the University College Dublin Human Research Ethics Committee on February 4, 2024 (LS-23-66). This study’s protocol was designed in accordance with the Towards Intelligent Health and Well-Being Network of Physical Activity Assessment (INTERLIVE) consortium’s best practice recommendations for validation of consumer wearables.^19^

### Generating VO_2_ max Estimates With Apple Watch

Apple Watch generates VO_2_ max estimates, proprietarily known as *Cardio Fitness*, by assessing an individual’s heart rate response to outdoor walking, running, or hiking activities on ground of less than 5% incline or decline.^10^ Adequate GPS and heart rate signal are required, alongside an increase of approximately 30% in heart rate from the resting value.^10^ Multiple activities are required to generate an estimate, although this number varies between users.^10^ Participants were informed of the required procedure and asked to generate an estimate independently within 1 week of criterion testing, in accordance with manufacturer guidelines.^10^ Before completing any activity, all required demographic information was entered in the Health app—including height, weight, sex, and age. Each participant was provided with an Apple Watch Series 10, which they were instructed to wear until at least 1 VO_2_ max estimate was generated. Each Apple Watch was running watchOS 11, ensuring that this study exclusively assessed Apple’s most recent VO_2_ max prediction algorithm and software version.

### Indirect Calorimetry Testing

Each participant completed a maximal cardiopulmonary exercise test (CPET) using indirect calorimetry.^20^ An exercise treadmill test was conducted in accordance with the American College of Sports Medicine’s Guidelines for Exercise Testing and Prescription, following the modified Åstrand protocol.^6^^,^^21^ A treadmill speed between 8 and 13 km/h was selected; this remained consistent throughout.^21^ The incline of the treadmill was increased by 2.5% every 2 minutes, following an initial 3-minute warm-up period at 0% incline.^21^ Participants were instructed to refrain from caffeine and nicotine for 12 hours before testing and to avoid strenuous activity and alcohol for a minimum of 24 hours.^6^ The COSMED Quark CPET metabolic cart (COSMED) was calibrated before each test according to the manufacturer’s instructions, and testing was conducted on the h/p/cosmos Venus treadmill (h/p/cosmos).

To ensure that VO_2_ max had been attained, participants were required to meet at least 2 of the following criteria: heart rate within ±10 bpm of age-predicted maximum (220 – age), respiratory exchange ratio ≥1.15, rate of perceived exertion ≥17, and VO_2_ plateau, defined as an increase in VO_2_ of <150 mL/min, with an increase in work rate as evidenced by physiological data.^6^ If at least 2 of these criteria were not met, the value was regarded as a VO_2_ peak. Participants who did not attain VO_2_ max were not included in the final analysis.

After the test, breath-by-breath data from the COSMED Quark CPET were filtered using a 30-second moving time average and exported to Microsoft Excel (Microsoft Corporation). The highest time-averaged VO_2_/kg value was interpreted as VO_2_ max. This was compared with the most recent Apple Watch VO_2_ max estimate available in the Health app.

### Outcomes

The primary outcome was the agreement between VO_2_ max estimates from Apple Watch and VO_2_ max values obtained via indirect calorimetry. This was calculated using Bland-Altman limits of agreement (LoA) analysis,^22^ mean absolute percentage error, and mean absolute error (MAE).

### Statistical Analyses

The target sample size was calculated based on the sample sizes of previous studies which validated VO_2_ max estimates from Apple Watch.^17^^,^^18^ The Bland-Altman 95% LoA were defined as the mean difference ±1.96 times the SD of the differences. The mean difference (bias) was calculated as the values from Apple Watch minus the criterion measure. A Bland-Altman plot was generated to visually represent the agreement. Mean absolute percentage error and MAE were also calculated. The t-critical values were used to compute corresponding 95% CI to account for the sample size. Analyses were conducted in Python (version 3.13.7) using the pandas, Matplotlib, and NumPy packages. The code used is available at github.com/rorylambe/applewatch-validation.

## Results

### Baseline Characteristics

Forty individuals participated in this study, and 35 were included in the final analysis (mean age [SD], 22.7 [2.9] years; 51% women). Of the 40 participants, 3 men and 2 women participants could not be included in the analysis: 1 participant failed to attain the criteria for VO_2_ max during CPET, and 4 participants did not generate a VO_2_ max estimate with Apple Watch within 1 week of criterion testing due to insufficient device wear and activity. Each participant’s cardiorespiratory fitness level, determined by indirect calorimetry testing, was classified in accordance with the Fitness Registry and the Importance of Exercise National Database (FRIEND) percentiles.^23^ Twenty-five (71%) participants were in the 80th percentile or higher, and 5 (14%) were in the 60th percentile or lower. Demographic characteristics of the participants included in the final analysis are listed in Table 1.

**Table 1:** Demographic Characteristics of Participants at Baseline^a^

Characteristic	Value
Age (y), mean (SD)	22.66 (2.93)
Age range (y)	20-36
Female, n	18 (51%)
Height (cm), mean (SD)	174.0 (9.80)
Weight (kg), mean (SD)	72.92 (11.00)
BMI (kg/m^2^), mean (SD)	23.98 (2.24)
Fitzpatrick skin tone^b^	
Type I	2 (0.1%)
Type II	12 (34%)
Type III	16 (45%)
Type IV	5 (14%)
CRF percentile, n^c^	
90th	14 (40%)
80th	11 (31%)
70th	5 (14%)
60th	3 (0.1%)
50th	0 (0%)
40th	1 (0.03%)
30th	1 (0.03%)

a Abbreviations: BMI, body mass index; CRF, cardiorespiratory fitness.

b Number of participants of each skin tone according to the Fitzpatrick scale.

c Number of participants of each cardiorespiratory fitness level classified according to the Fitness Registry and the Importance of Exercise National Database (FRIEND).

### Apple Watch Agreement With the Criterion

Apple Watch Series 10 underestimated VO_2_ max in comparison with indirect calorimetry. The mean difference was –6.25 mL/kg per minute (SD, 6.11; 95% CI, –8.28 to –4.23). Bland-Altman LoA indicated variability between the two measurement methods (–18.23 to 5.73; Figure). The mean absolute percentage error was 13.2%, and the MAE was 6.79 mL/kg per minute. Apple Watch correctly classified the FRIEND cardiorespiratory fitness percentile of 5 (14%) participants and classified a further 12 (34%) within one adjacent percentile band of their actual band. A total of 18 (51%) participants were misclassified by two percentile bands or more. The results are summarized in Table 2.

**Figure:**
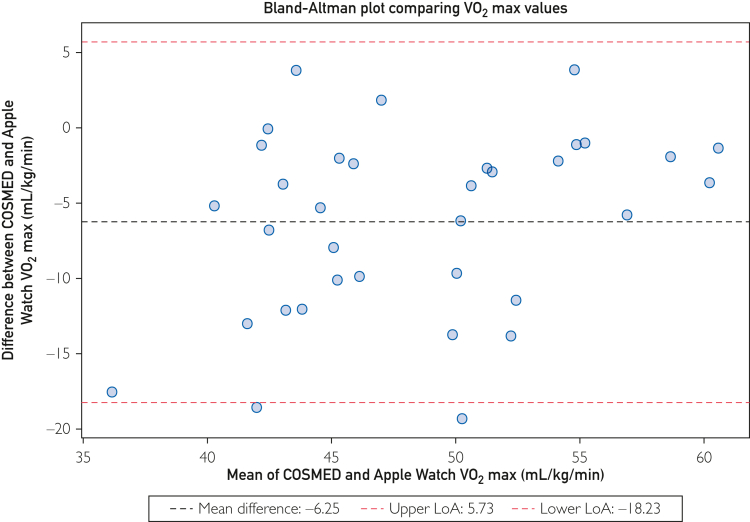
Bland-Altman plot illustrating the agreement between VO_2_ max estimates from Apple Watch and measurements obtained via indirect calorimetry using the COSMED Quark metabolic cart. LoA, limits of agreement.

**Table 2:** Summary of Results^a^^,^^b^

Statistical measure	Result
Mean (SD) (mL/kg per min)	
Apple Watch	45.26 (7.64)
COSMED^a^	51.51 (5.88)
Standard error of the mean (mL/kg per min)	
Apple Watch	1.29
COSMED	0.99
Standard deviation of the differences (mL/kg per min)	6.11
Mean difference (95% CI) (mL/kg per min)	–6.25 (–8.28 to –4.23)
Bland-Altman limits of agreement (mL/kg per min)	
Lower limit of agreement (95% CI)	–18.23 (–21.73 to –14.74)
Upper limit of agreement (95% CI)	5.73 (2.24-9.23)
Mean absolute percentage error (95% CI)	13.2 (9.6-16.8)
Mean absolute error (95% CI) (mL/kg per min)	6.79 (4.91-8.67)

a Abbreviation: COSMED, COSMED Quark cardiopulmonary exercise testing metabolic cart.

b Statistical agreement between Apple Watch and indirect calorimetry.

### Exploratory Subgroup Analyses by Sex and Cardiorespiratory Fitness

We conducted post hoc exploratory subgroup analyses to examine whether agreement differed by sex or cardiorespiratory fitness level. Bland-Altman analysis showed larger mean difference and wider LoA for women (n=18; mean difference, –8.55 mL/kg per minute [95% CI, –11.58 to –5.51]; LoA, –21.41 to 4.32) than for men (n=17; mean difference, –3.82 [95% CI, –6.02 to –1.62]; LoA, –12.90 to 5.26). Notably, a larger proportion of female than male participants were in the ≥80th percentile of cardiorespiratory fitness (16 of 18 [89%] vs 9 of 17 [53%]).

To explore potential differences by the cardiorespiratory fitness level, we classified participants into 2 distinct subgroups: higher fitness (≥80th percentile, n=25), and lower fitness (≤70th percentile, n=10). These thresholds were chosen to compare individuals with very high cardiorespiratory fitness to those with moderate-to-poor fitness, while maintaining a sufficient subgroup size for meaningful analysis. We found a larger mean difference and wider LoA for participants with high cardiorespiratory fitness (lower fitness: mean difference –2.41 mL/kg per minute [95% CI, –5.28 to 0.47], LoA, –11.51 to 6.69; higher fitness: mean difference –7.79 mL/kg per minute [95% CI, –10.15 to –5.43], LoA, –19.59 to 4.01; Table 3). Multiple linear regression indicated that sex and fitness together explained 22.6% of the variance in mean difference (*R*^*2*^=0.226; adjusted *R*^*2*^=0.178; F(2, 32)=4.68; *P*=.016; n=35). However, neither sex (B=–3.32, 95% CI, –7.48 to 0.85; *P*=.11) nor fitness level (B=–3.92, 95% CI, –8.53 to 0.68; *P*=.09) was independently associated with mean difference. These results should be considered hypothesis-generating, as subgroup sizes were small. Additional details of subgroup analyses, including Bland-Altman plots, are available in Supplemental Tables 1-6 and Supplemental Figures 1-4, available online at https://www.mcpdigitalhealth.org/.

**Table 3:** Summary of Subgroup Analysis Results^a^^,^^b^

Statistical measure	Higher CRF (≥80th %)	Lower CRF (≤70th %)	Male	Female
No. of participants	25	10	17	18
Mean (SD) (mL/kg per min)				
Apple Watch	44.95 (8.37)	46.03 (5.70)	50.65 (6.11)	40.17 (4.99)
COSMED	52.74 (5.95)	48.44 (4.66)	54.47 (5.34)	48.71 (5.03)
SEM (mL/kg per min)				
Apple Watch	1.67	1.80	1.48	1.18
COSMED	1.19	1.47	1.30	1.18
SD_diff_ (mL/kg per min)	6.02	4.64	4.63	6.56
Mean difference (95% CI) (mL/kg per min)	–7.79 (–10.15 to –5.43)	–2.41 (–5.28 to 0.47)	–3.82 (–6.02 to –1.62)	–8.55 (–11.58 to –5.51)
Bland-Altman LoA (mL/kg per min)				
Lower LoA (95% CI)	–19.59 (–23.68 to –15.50)	–11.51 (–16.60 to –6.41)	–12.90 (–16.74 to –9.06)	–21.41 (–26.69 to –16.13)
Upper LoA (95% CI)	4.01 (–0.08 to 8.10)	6.69 (1.60-11.79)	5.26 (1.42-9.10)	4.32 (–0.96 to 9.60)
MAPE (95% CI) (%)	14.89 (10.10-19.69)	8.87 (4.98-12.76)	8.19 (4.59-11.78)	17.88 (12.28-23.49)
MAE (95% CI) (mL/kg per min)	7.78 (5.30-10.27)	4.31 (2.37-6.25)	4.49 (2.46-6.52)	8.97 (6.02-11.92)

a Abbreviations: COSMED, COSMED Quark cardiopulmonary exercise testing metabolic cart; CRF, cardiorespiratory fitness; LoA, 95% limits of agreement; MAE, mean absolute error; MAPE, mean absolute percentage error; SD_diff_, standard deviation of the differences; SEM, standard error of the mean.

b Statistical agreement between Apple Watch and indirect calorimetry between cardiorespiratory fitness and sex subgroups.

## Discussion

In this study, we validated VO_2_ max estimates from Apple Watch Series 10 against indirect calorimetry among a sample predominantly consisting of individuals with high cardiorespiratory fitness. We found that Apple Watch underestimated VO_2_ max, with a clinically significant mean difference and LoA (–6.25 [95% CI, –8.28 to –4.23] mL/kg per minute; LoA, –18.23 to 5.73 mL/kg per minute).

In line with the recommendations of Bland and Altman, the LoA are the key measure to determine measurement performance relative to the criterion, whereas mean difference reflects systematic positive or negative bias.^24^ The error we observed substantially exceeded the typical error of indirect calorimetry, estimated as ±5% (mean standard error of 2.58 mL/kg per minute).^25^^,^^26^ However, the accuracy aligned more closely with traditional submaximal predictions; LoA of approximately ±11 mL/kg per minute have been previously reported.^7^, ^8^, ^9^

In our subgroup analyses, we found a smaller mean difference and narrower LoA among participants in the ≤70th FRIEND cardiorespiratory fitness percentile (mean difference, –2.41 mL/kg per minute; LoA, –11.51 to 6.69). Although these results are exploratory and hypothesis-generating, they highlight the need to examine demographic and physiological characteristics that affect measurement accuracy.^27^

Many factors affect measurement accuracy, influenced both by Apple’s prediction algorithm and procedure. First, the high degree of individual variation in heart rate response to exercise complicates the extrapolation required to generate estimates.^28^ Although lower heart rate during exercise typically indicates higher cardiorespiratory fitness, this response varies with training type and genetic factors. Second, lower-intensity exercise, often used to inform Apple Watch estimates, requires additional extrapolation of an individual’s physiological response compared with exercise close to maximal exertion. Third, VO_2_ max is estimated based on inputs from multiple motion sensors, photoplethysmography waveforms, demographic information, and GPS-derived metrics—such as speed, distance, and elevation. Error from individual inputs may compound when they are combined to generate a prediction.^29^ Fourth, when environmental conditions—such as terrain, gradient, or heat—increase exercise workload, Apple Watch may misinterpret the user’s physiological response. Lacking the contextual information that corresponds with increased workload, the device may inaccurately classify an elevated heart rate as a sign of poor cardiorespiratory fitness, rather than a reflection of the actual effort. Similarly, intake of caffeine, alcohol or medications that affect heart rate also influences estimates. Considering these complexities, the uncontrolled exercise that informs estimates increases the level of uncertainty and error.

To determine whether accuracy is adequate, the measurement’s intended use should be considered. For clinical use, thresholds of clinically meaningful change—related to disease risk, for instance—may guide implementation. For example, each 1-Metabolic Equivalent of Task (MET) (3.5 mL/kg per minute VO_2_) increase in cardiorespiratory fitness is associated with an 11% decrease in all-cause mortality risk.^1^ In this context, the mean difference we found (–6.25 mL/kg per minute) could meaningfully alter clinical risk interpretation, shifting an individual by 2 to 3 FRIEND percentile bands. For general fitness monitoring, wider margins of error may provide high-level time trends in cardiorespiratory fitness. Wider error margins may also be adequate for population-level assessment or in large research trials, as individual error is attenuated by scale.

Our findings were comparable to those previously reported for Apple Watch and devices from other manufacturers, although there are some important distinctions.^17^^,^^18^ Although previous validation of Apple Watch reported similar LoA, this literature is limited by sample size and the rigor of statistical and procedural protocols, including comparison of VO_2_ max values obtained during different exercise modes (eg, cycling vs running/walking), a method that is not recommended. In addition, the use of criterion measures other than indirect calorimetry restricts the validity of past findings.^30^ Elsewhere, a meta-analysis that pooled results from Garmin, Polar, and Fitbit devices found a small mean bias but wide LoA.^19^^,^^31^^,^^32^ Together, these findings indicate low systemic bias from wearable-derived VO_2_ max but poor validity of individual estimates.

Although this restricts the utility of measurements obtained at single time points, the relationship of Apple Watch estimates with changes in VO_2_ max over time remains unclear. Traditional submaximal predictions are clinically valuable as they provide a reliable method of measurement, rather than a valid one.^6^ This reveals longitudinal trends in cardiorespiratory fitness and is used to monitor rehabilitation of patients. Yet, to the best of our knowledge, there has been no independent validation of Apple Watch measurement reliability to date. Similarly, we were unable to assess reliability due to our cross-sectional design.

Our study has several limitations. Most notably, our sample was predominantly young, healthy, and had high cardiorespiratory fitness, with only 2 participants classified in the 40th percentile or lower. This substantially limits the generalizability of our findings to individuals with low cardiorespiratory fitness and clinical populations. The size and composition of our sample restricted subgroup analyses and exploration of confounders. Apple’s proprietary machine learning algorithm further limited interpretability. Consequently, our subgroup analyses are hypothesis-generating, rather than confirmatory. In addition, we did not formally quantify habitual physical activity, restricting characterization of behavioral factors that may have influenced VO_2_ max estimation.

The study has a number of strengths. Our procedural and statistical methodology was robust, guided by the INTERLIVE consortium’s expert statement.^19^ Apple Watch estimates were validated against the gold standard method, and a sex-balanced sample was recruited. Participants generated VO_2_ max estimates in a free-living environment, which best reflects typical device use and enhances the ecological validity of our findings. Notably, this is the first study to validate Apple Watch Series 10, which uses the latest VO_2_ max prediction algorithm and optical heart rate sensor.^10^^,^^33^

As Apple Watch software and hardware develop, it may complement conventional VO_2_ max prediction methods. The accuracy of conventional predictions varies considerably based on factors outlined by the American College of Sports Medicine, which include the formulas used to predict maximum heart rate, premature cessation of exercise testing by patients, and deviation from a linear heart rate-workload relationship.^6^, ^7^, ^8^, ^9^^,^^34^, ^35^, ^36^ Additionally, conventional predictions are often sporadic as they require clinician oversight. In contrast, the continuous physiological data used to inform Apple Watch VO_2_ max estimates, alongside potential personalization from machine learning approaches, may be advantageous. Incorporating a standardized testing procedure—requiring users to input specific information and conduct a particular type of exercise—may reduce the impact of confounding variables and improve accuracy.

Continued validation is required to establish the utility of Apple Watch estimates. Apple last updated its prediction algorithm in 2021, with 534 individuals contributing to development.^10^ Larger, more diverse training datasets may enable algorithms that account for physiological variability with more nuance, adapting prediction to individual users. Future validation should include older adults and those with low cardiorespiratory fitness, and the subgroup patterns we observed warrant investigation in larger samples. Contextual factors that influence prediction accuracy, such as variation in physiological response to exercise, should also be investigated.

## Conclusion

Our findings demonstrate that Apple Watch underestimated VO_2_ max, with substantial variability in measurement error limiting the clinical use of individual estimates. In large clinical trials, however, Apple Watch enables scalable digital end point collection at the population-level. Validation of measurement reliability is needed to determine its suitability for monitoring longitudinal changes in cardiorespiratory fitness.

## Potential Competing Interests

The authors report no competing interests.

## Ethics Statement

Ethical approval was granted by the University College Dublin Human Research Ethics Committee on February 4, 2024 (LS-23-66).
