# Supplementary material for: Accuracy of VO2 max Estimates From Apple Watch Series 10
Source: Mayo Clin Proc Digit Health. 2026 Apr 7;4(2):100357. doi: 10.1016/j.mcpdig.2026.100357 (PMC13141568; doi:10.1016/j.mcpdig.2026.100357)
Supplement: Supplemental Appendix [file mmc1.docx]

**Accuracy of VO_2_ max estimates from Apple Watch Series 10.**

**Supplemental Appendix**

**Table of contents**

Demographic characteristics of all participants p. 1

Results of subgroup analysis by sex pp. 2–3

Bland-Altman limits of agreement plots (male, female) p. 4

Results of subgroup analysis by cardiorespiratory fitness level pp. 5–6

Bland-Altman limits of agreement plots (male, female) p. 7

Results of multiple linear regression p. 8

**Demographic characteristics of all recruited participants.**

**TABLE S1.** Characteristics of all 40 participants.

| **Characteristic** | **Value** |
| --- | --- |
| Age, mean (SD), years | 22.52 (2.80) |
| Female, no. (%) | 20 (50%) |
| Height, mean (SD), cm | 174.36 (9.42) |
| Weight, mean (SD), kg | 72.59 (10.57) |
| BMI, mean (SD), kg/m^2^ | 23.98 (2.24) |
| Fitzpatrick skin tone ^a^ |  |
| Type I | 2 |
| Type II | 14 |
| Type III | 19 |
| Type IV | 5 |
| CRF percentile ^b^ |  |
| 90^th^, no. | 16 |
| 80^th^, no. | 13 |
| 70^th^, no. | 5 |
| 60^th^, no. | 4 |
| 50^th^, no. | 0 |
| 40^th^, no. | 1 |
| 30^th^, no. | 1 |

**BMI:** Body Mass Index

^a^ Number of participants of each skin tone according to the Fitzpatrick Scale.

^b^ Number of participants of each cardiorespiratory fitness level classified according to FRIEND.

**Subgroup analysis including male participants only**

**TABLE S2.** Statistical agreement between Apple Watch and indirect calorimetry.

| **Statistical measure** | **Result** |
| --- | --- |
| Total no. participants | 17 |
| Mean (SD), mL/kg/min |  |
| Apple Watch | 50.65 (6.11) |
| COSMED | 54.47 (5.34) |
| Standard error of the mean, mL/kg/min |  |
| Apple Watch | 1.48 |
| COSMED | 1.30 |
| Standard deviation of the differences, mL/kg/min | 4.63 |
| Mean difference (95% CI), mL/kg/min | -3.82 (-6.02 to -1.62) |
| Bland-Altman limits of agreement, mL/kg/min |  |
| Lower limit of agreement (95% CI) | -12.90 (-16.74 to -9.06) |
| Upper limit of agreement (95% CI) | 5.26 (1.42 to 9.10) |
| Mean absolute percentage error (95% CI) | 8.19% (4.59 to 11.78) |
| Mean absolute error, mL/kg/min (95% CI) | 4.49 (2.36 to 6.52) |

* COSMED = COSMED Quark CPET metabolic cart.

**Subgroup analysis including female participants only**

**TABLE S3.** Statistical agreement between Apple Watch and indirect calorimetry.

| **Statistical measure** | **Result** |
| --- | --- |
| Total no. participants | 18 |
| Mean (SD), mL/kg/min |  |
| Apple Watch | 40.17 (4.99) |
| COSMED | 48.71 (5.03) |
| Standard error of the mean, mL/kg/min |  |
| Apple Watch | 1.18 |
| COSMED | 1.18 |
| Standard deviation of the differences, mL/kg/min | 6.56 |
| Mean difference (95% CI), mL/kg/min | -8.55 (-11.58 to -5.51) |
| Bland-Altman limits of agreement, mL/kg/min |  |
| Lower limit of agreement (95% CI) | -21.41 (-26.69 to -16.13) |
| Upper limit of agreement (95% CI) | 4.32 (-0.96 to 9.60) |
| Mean absolute percentage error (95% CI) | 17.88% (12.28 to 23.49) |
| Mean absolute error, mL/kg/min (95% CI) | 8.97 (6.02 to 11.92) |

* COSMED = COSMED Quark CPET metabolic cart.

**
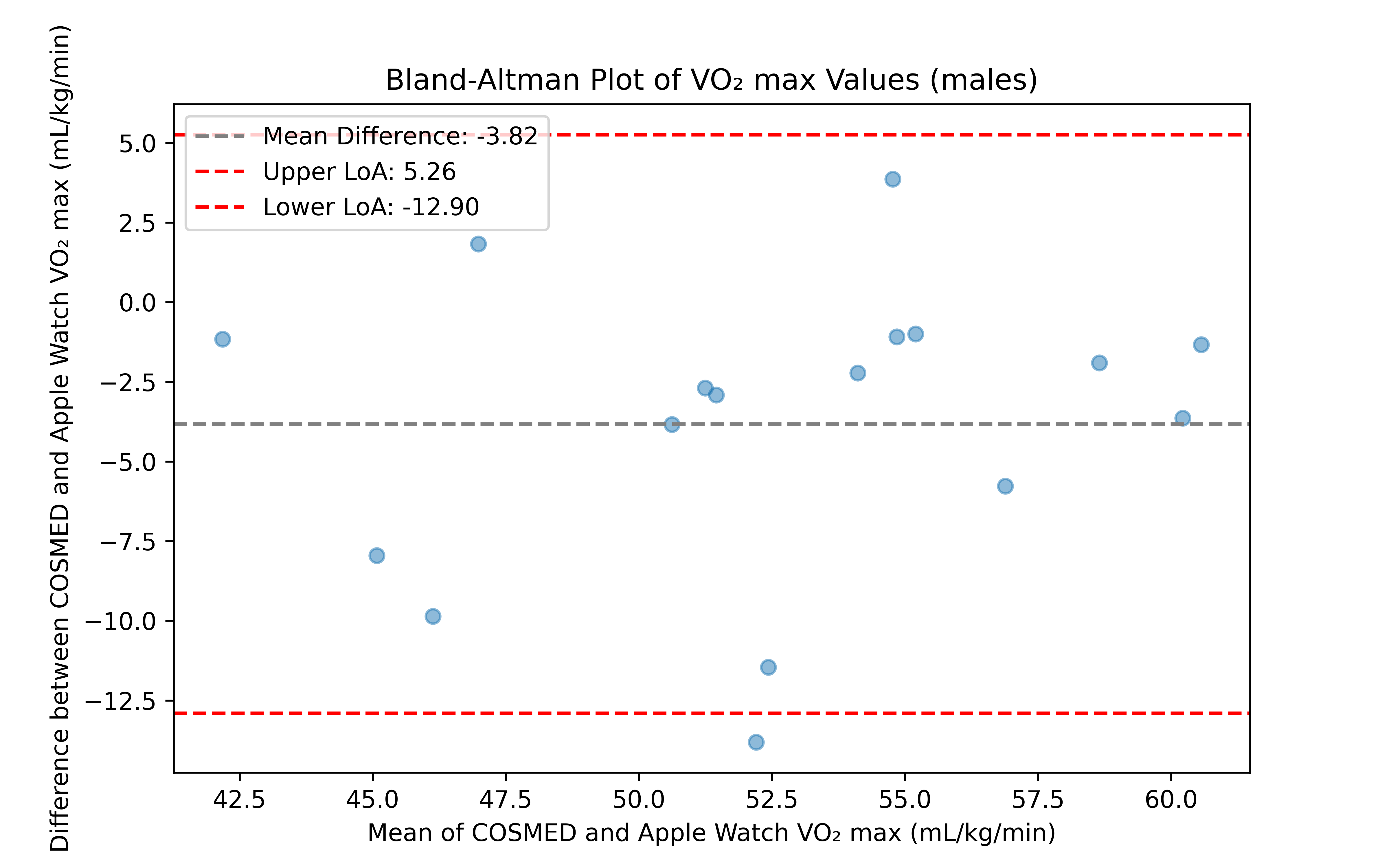
**

**FIGURE S1.** Bland-Altman plot illustrating the agreement between Apple Watch and indirect calorimetry, among male participants only.

**
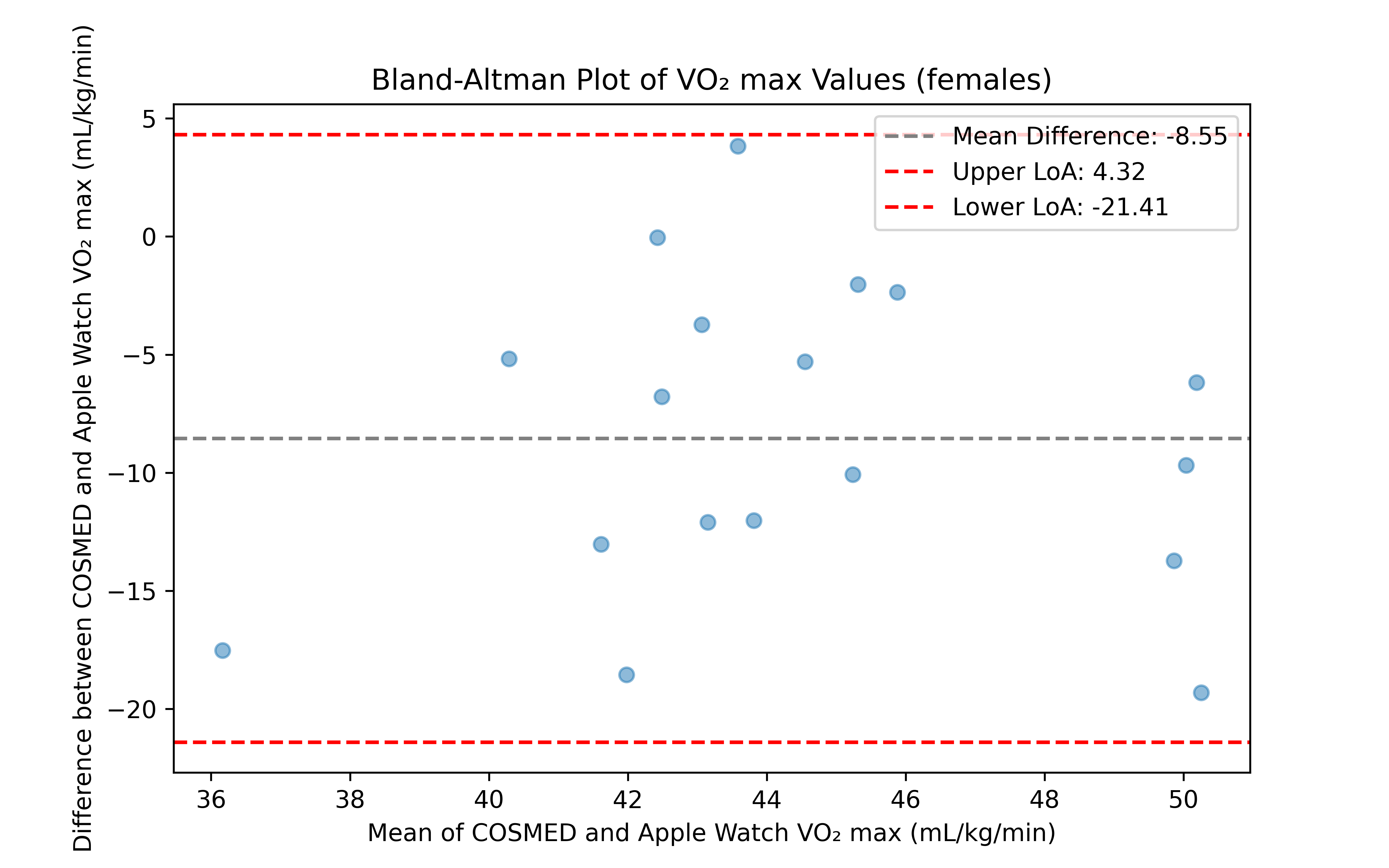
**

**FIGURE S2.** Bland-Altman plot illustrating the agreement between Apple Watch and indirect calorimetry, among female participants only.

**Subgroup analysis including participants with cardiorespiratory fitness in the 80^th^ percentile, or higher.**

**TABLE S4.** Statistical agreement between Apple Watch and indirect calorimetry.

| **Statistical measure** | **Result** |
| --- | --- |
| Total no. participants | 25 |
| Mean (SD), mL/kg/min |  |
| Apple Watch | 44.95 (8.37) |
| COSMED | 52.74 (5.95) |
| Standard error of the mean, mL/kg/min |  |
| Apple Watch | 1.67 |
| COSMED | 1.19 |
| Standard deviation of the differences, mL/kg/min | 6.02 |
| Mean difference (95% CI), mL/kg/min | -7.79 (-10.15 to -5.43) |
| Bland-Altman limits of agreement, mL/kg/min |  |
| Lower limit of agreement (95% CI) | -19.59 (-23.68 to -15.50) |
| Upper limit of agreement (95% CI) | 4.01 (-0.08 to 8.10) |
| Mean absolute percentage error (95% CI) | 14.89% (10.10 to 19.69) |
| Mean absolute error, mL/kg/min (95% CI) | 7.79 (5.30 to 10.27) |

* COSMED = COSMED Quark CPET metabolic cart.

**Subgroup analysis including participants with cardiorespiratory fitness in the 70^th^ percentile, or lower.**

**TABLE S5.** Statistical agreement between Apple Watch and indirect calorimetry.

| **Statistical measure** | **Result** |
| --- | --- |
| Total no. participants | 10 |
| Mean (SD), mL/kg/min |  |
| Apple Watch | 46.03 (5.70) |
| COSMED | 48.44 (4.66) |
| Standard error of the mean, mL/kg/min |  |
| Apple Watch | 1.80 |
| COSMED | 1.47 |
| Standard deviation of the differences, mL/kg/min | 4.64 |
| Mean difference (95% CI), mL/kg/min | -2.41 (-5.28 to 0.47) |
| Bland-Altman limits of agreement, mL/kg/min |  |
| Lower limit of agreement (95% CI) | -11.51 (-16.60 to -6.41) |
| Upper limit of agreement (95% CI) | 6.69 (1.60 to 11.79) |
| Mean absolute percentage error (95% CI) | 8.87% (4.98 to 12.76) |
| Mean absolute error, mL/kg/min (95% CI) | 4.31 (2.37 to 6.25) |

* COSMED = COSMED Quark CPET metabolic cart.

**
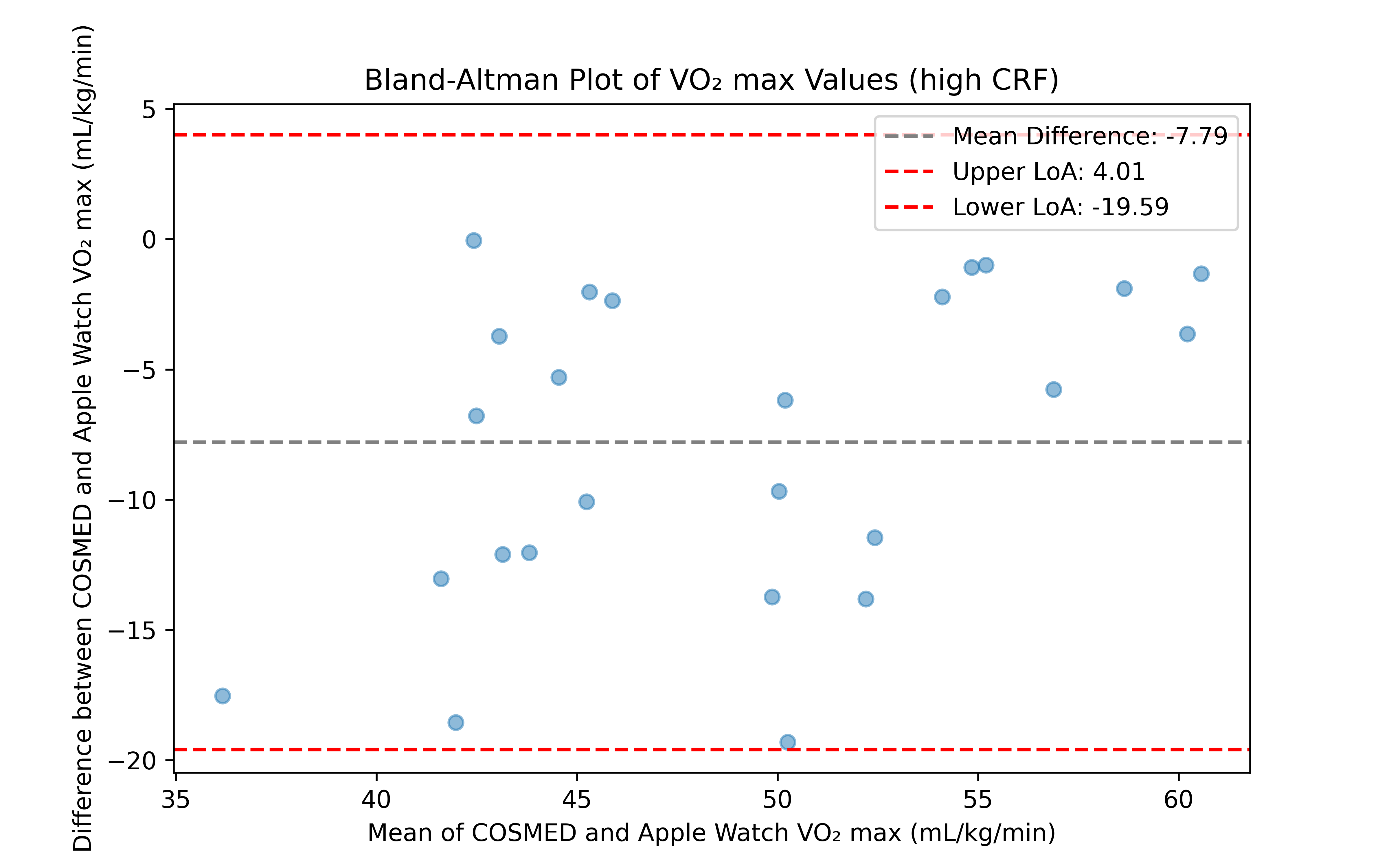
**

**FIGURE S3.** Bland-Altman plot illustrating the agreement between Apple Watch and indirect calorimetry, among participants in the ≥80th FRIEND cardiorespiratory fitness percentile.

**
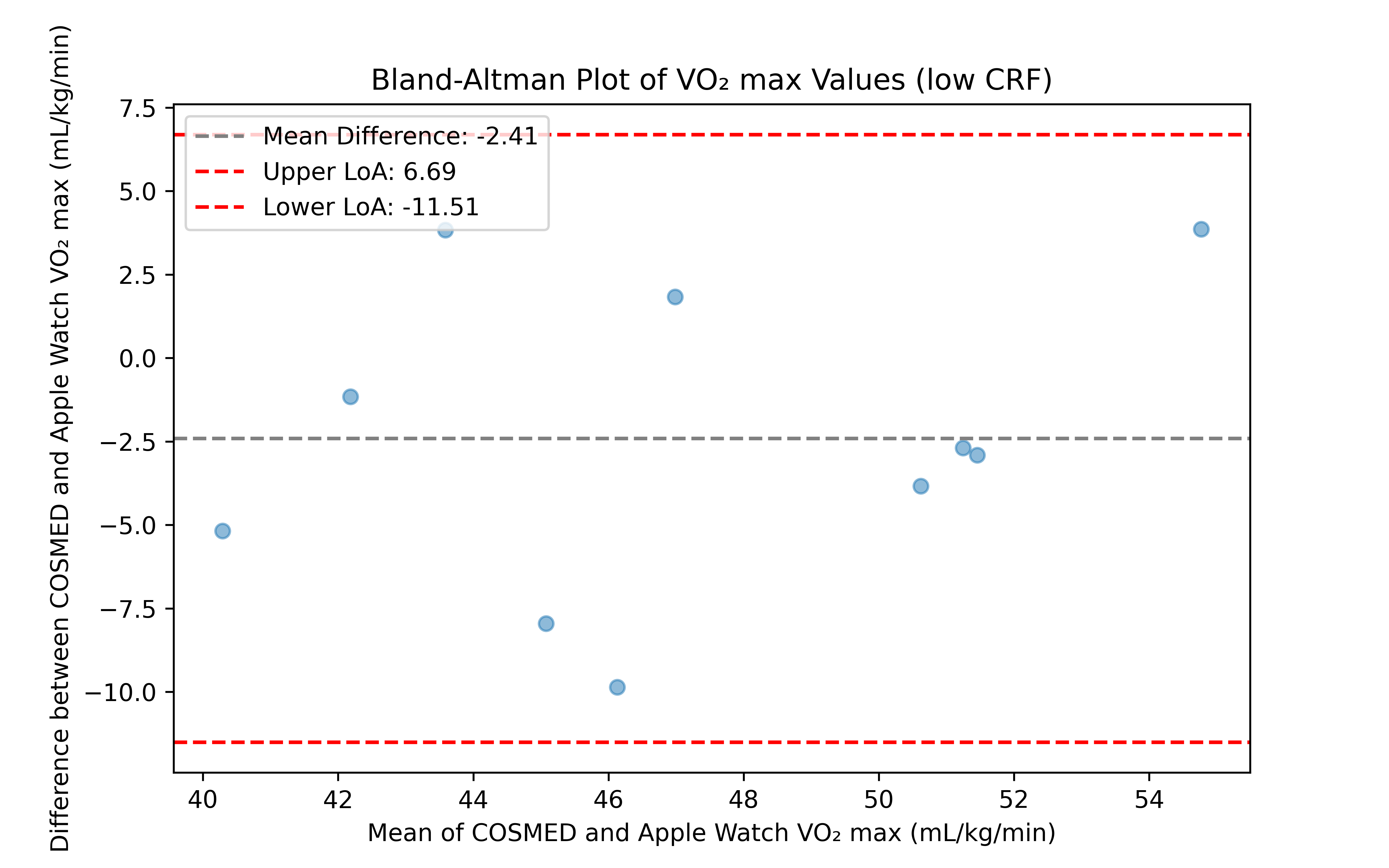
**

**FIGURE S4.** Bland-Altman plot illustrating the agreement between Apple Watch and indirect calorimetry, among participants in the ≤70th FRIEND cardiorespiratory fitness percentile.

**Ordinary Least Squares Regression Results**

| Variable | coef | std err | t | P>\|t\| | 95% CI lower | 95% CI upper |
| --- | --- | --- | --- | --- | --- | --- |
| const | -1.744 | 1.800 | -0.969 | 0.340 | -5.410 | 1.922 |
| sex | -3.316 | 2.043 | -1.623 | 0.114 | -7.478 | 0.845 |
| crf | -3.922 | 2.260 | -1.735 | 0.092 | -8.526 | 0.682 |

**TABLE S6. Ordinary least squares regression results.** CRF, cardiorespiratory fitness percentile classified according to FRIEND 2022 reference standards (RER ≥ 1.0).
